# Development and Evaluation of the Properties of Active Films for High-Fat Fruit and Vegetable Packaging

**DOI:** 10.3390/molecules28073045

**Published:** 2023-03-29

**Authors:** Cristina Muñoz-Shugulí, Francisco Rodríguez-Mercado, Nasreddine Benbettaieb, Abel Guarda, María José Galotto, Frederic Debeaufort

**Affiliations:** 1 Packaging Innovation Center (LABEN), University of Santiago of Chile (USACH), Santiago 9170201, Chile; 2 Center for the Development of Nanoscience and Nanotechnology (CEDENNA), University of Santiago of Chile (USACH), Santiago 9170022, Chile; 3 Facultad de Ciencias, Escuela Superior Politécnica de Chimborazo (ESPOCH), Riobamba 060106, Ecuador; 4 Food Processing and Microbiology Joint Unit UMR PAM, l’Institut AgroDijon, University of Bourgogne Franche-Comté, 21000 Dijon, France; 5 Department of Bioengineering, IUT-Dijon-Auxerre, University of Burgundy, CEDEX, 20178 Dijon, France

**Keywords:** poly(lactic acid), allyl isothiocyanate, cyclodextrin, inclusion complex, surface properties, active compound release, Fickian diffusion, active food packaging

## Abstract

β-cyclodextrin and allyl isothiocyanate inclusion complexes (β-CD:AITC) have been proposed for developing fresh fruit and vegetable packaging materials. Therefore, the aim of this research was to develop active materials based on poly(lactic acid) (PLA) loaded with β-CD:AITC and to assess changes in the material properties during the release of AITC to food simulants. PLA films with 0, 5 and 10 wt.% β-CD:AITC were developed by extrusion. Surface properties were determined from contact angle measurements. Films were immersed in water, aqueous and fatty simulants to assess the absorption capacity and the change in the thermal properties. Moreover, the release of AITC in both simulants was evaluated by UV-spectroscopy and kinetic parameters were determined by data modeling. Results showed that a higher concentration of β-CD:AITC increased the absorption of aqueous simulant of films, favoring the plasticization of PLA. However, the incorporation of β-CD:AITC also avoided the swelling of PLA in fatty simulant. These effects and complex relationships between the polymer, inclusion complexes and food simulant explained the non-systematic behavior in the diffusion coefficient. However, the lower partition coefficient and higher percentage of released AITC to the fatty simulant suggested the potential of these materials for high-fat fruit and vegetable active packaging applications.

## 1. Introduction

The growth of undesirable microorganisms in fruit and vegetables is an important issue, since they reduce the food shelf-life and increase the risk to consumers of foodborne illness. To confront this problem, the food industry has developed various technologies such as thermal processing, drying, freezing, refrigeration, irradiation and modified atmosphere [1]. However, these technologies cannot be applied in certain products. Therefore, the introduction of active agents with antimicrobial activity in the packaging system has been considered as an interesting alternative for improving food safety [2].

It is well known that antimicrobial packaging interacts with packaged food in order to reduce, retard or inhibit the growth of spoilage and pathogenic microorganisms [3]. A wide range of antimicrobial substances could be used for developing active food packaging systems. However, those derived from natural products have been of great interest in recent years. Essential oils and their components extracted from many spices, plants and fruits have been recognized as antibacterial and antifungal compounds against a broad spectrum of microorganisms [4]. In addition, essential oils are generally recognized as safe (GRAS) by the U.S Food and Drug Administration (FDA) and they have been registered by the European Commission as presenting no risk to the health of customers, which supports their use in the development of active food packaging [5].

In this regard, allyl isothiocyanate (AITC, 3-isothiocyanato-1-propene), the major antimicrobial component of the essential oil from black and brown mustard seeds, has shown excellent potential to control the growth of different microorganism at low concentrations. For instance, AITC at 0.07 to 2 ppm has been used as postharvest treatment for preventing mold and yeast growth in foods with high water content, such us strawberries and apples [6,7]. AITC has also been applied for preventing the growth of bacteria in fatty food. Reduction of *Pseudomonas lundensis*, *Staphylococcus aureus*, *Bacillus cereus* and aerobic mesophilic counts in chicken meat have been achieved using 1000 ppm of AITC in combination with vacuum packaging [8]. Similarly, AITC (0.05% *w*/*w*) combined with acetic acid and high pressure processing was shown to be effective for preventing *Salmonella* growth in chicken [9]. Moreover, 0.5% or 1% (*v*/*w*) of AITC has been used for cottage cheese preservation and 1 mg.100 mg^−1^ of AITC prevented yeast and mold growth in mozzarella cheese [10]. Nevertheless, the main disadvantage of using AITC is its highly volatile nature, which limits its application in active food packaging [11]. To address this challenge, recent studies have reported the effectiveness of cyclodextrins to encapsulate essential oils, providing a slow release of these compounds [12].

Within the different types of cyclodextrins, beta-cyclodextrin (β-CD) is the most accessible, the lowest-priced and the most used [13]. It is formed by 7 D-glucopyranoside units linked by α-1,4- glycosidic bonds that form a truncated conic structure. It has a hydrophilic external wall and a relatively hydrophobic inner cavity that allow it to complex (encapsulate) a wide variety of hydrophobic molecules, such as AITC [12]. The formation of inclusion complexes with β-CD allows the protection of the compound from thermal degradation, volatilization and, thus, decreases the guest release rate, which is one of the main challenges in the design of active food packaging materials [14]. A previous study demonstrated that the formation of β-CD:AITC inclusion complexes protected the active compound from thermal degradation [15]. In addition, it was observed that the release of AITC in the vapor phase increased with the relative humidity rise. This effect was related to the structure of the β-CD, which is changed by hydration. Moreover, β-CD:AITC inclusion complexes maintained the antimicrobial capacity of AITC at low concentrations (lower than 2 ppm). Inclusion complexes showed a fungicidal effect against *Botrytis cinerea*, a common decay mold in grapes, strawberries, blackberries, tomato and other fresh produce. Therefore, these agents were recommended for the design of active food packaging materials for fruit and vegetables.

Similarly to the active compounds, there is a trend towards using natural or bio-sourced materials that avoid plastic pollution. In this regard, poly(lactic acid) (PLA) is an important alternative to produce eco-friendly materials for the food industry [16]. To date, PLA has been the most used biopolymer because it is a compostable and exhibits particular properties, such as high rigidity, transparency and excellent processability [17]. However, incorporation of additives and the use of different processing technologies could change the properties of this polymer [18]. For instance, AITC has been incorporated as a bilayer PLA film thought an electrospinning coating technique [19]. Concentrations over 15 wt.% of AITC in relation to the PLA produced roughed beads in fibers (surface changes) and phase separation. On the other hand, Wang et al. [20] developed PLA/β-CD:AITC antimicrobial films by extrusion where no phase separation occurred. The incorporation of inclusion complexes improved the elongation at break, due to the increase of polymer chain mobility. This behavior influenced the release of AITC from these PLA films. The exposure of PLA materials to food and food simulants can also alter their structural properties. Since aqueous and fatty food simulants are the most representative, alcoholic solutions (10, 50 and 95% *v*/*v* ethanol solutions) are employed for assessing the release of active compounds from antimicrobial packaging. Sonchaeng et al. [21] reported a decrease of the glass–rubber transition temperature (T_g_) of casted PLA films after immersion in alcoholic solutions of different concentrations. It is well known that T_g_ are also related to the chain mobility (plasticization phenomena) and thus, with the release of active compounds from polymer films [18]. Therefore, it is important to understand how the changes in the structure and/or functional properties of PLA films by inclusion complexes and contact with different food simulants influence the release of AITC.

Based on the above, the aim of this work was to develop an active material based on PLA with β-CD:AITC inclusion complexes and to assess the changes in the material properties during the release of AITC to food simulants. Films loaded with β-CD:AITC inclusion complexes at 0, 5 and 10 wt.% were prepared and denoted as P0 (control), P5 and P10. This knowledge will allow the potential application of the materials for fruit and vegetable packaging to be determined.

## 2. Results and Discussion

### 2.1. Surface Properties

The evaluation of surface properties such as free energy (γ_S_) and critical surface energy (γ_c_) allowed qualitative determination of the hydrophobic/hydrophilic nature and affinity of the materials [22]. These parameters could be determined from contact angle (θ) measurements [23]. Moreover, the water contact angle (θ_w_) allows prediction of the hydration properties of materials [24], which is an important issue during active compound release.

The θ_w_ shows the attraction between water molecules and the material surface. Therefore, the lower it is, the stronger the attraction [25]. Quantitatively, when θ_w_ > 65° the surface is hydrophobic and when θ_w_ < 65°, this indicates a hydrophilic nature [26,27]. Furthermore, Yuan and Lee [28] stated that a wettable surface shows a θ_w_ < 90°.

Table 1 summarizes the θ_w_ obtained for PLA and active films. Values between 70 and 75° were observed for all samples. Thus, films could be considered as relatively hydrophobic but having a water-wettable surface. Furthermore, the incorporation of inclusion complexes to PLA significantly decreased the θ_w_ value. This change could be related to the partial exposure of inclusion complexes at the surface of materials, which are able to absorb water and, therefore, increase the film surface wettability.

On the other hand, the θ of PLA films with water (θ_w_), ethylene glycol (θ_EG_), diiodomethane (θ_DM_) and glycerol (θ_G_) were constant over time (120 s). However, the θ for ethanol was not quantifiable, since the solvent immediately spread in the film surfaces. Table 1 shows that materials have higher affinity with less polar solvent, since the values of θ followed the order θ_DM_ < θ_EG_ < θ_G_ < θ_w_, which confirmed the relatively hydrophobic surface of PLA. Likewise, θ_DM_ was lower and θ_EG_ was higher for P5 and P10 in comparison with P0, which shows an increase in the hydrophilicity of the film surface due to the presence of inclusion complexes.

**Figure 1:**
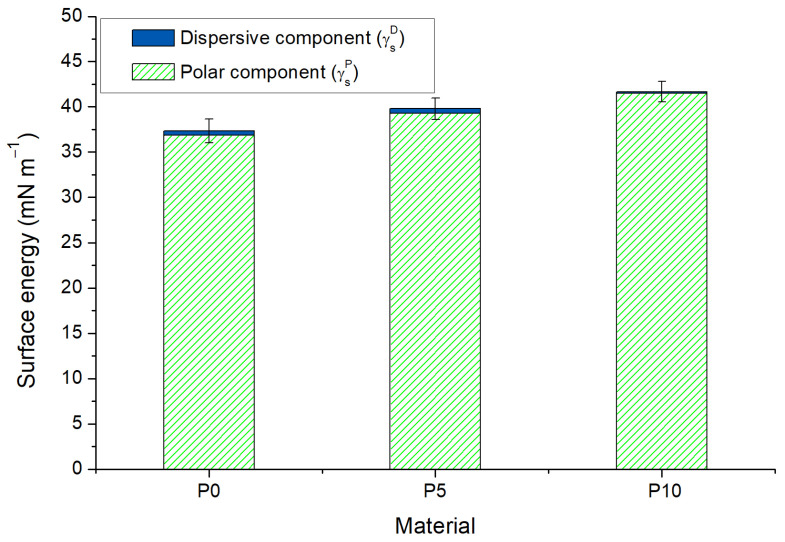
Surface energy and components of poly(lactic acid) (P0) and active films (P5 and P10). Error bars shows the standard deviation of total energy (γ_S_).

Free energy and its components were calculated using the θ measured with the four liquids. As Figure 1 shows, the free energy of P0 (γ_S_ = 37.3 ± 1.3 mN m^−1^) was dominated by the polar component (γ_S_^P^ > γ_S_^D^). This behavior is opposite to the data reported for PLA films in the literature, where γ_S_^P^ < γ_S_^D^ [29,30]. However, the high polarity of films was attributed to the free carboxyl groups that conduct to dipole–dipole interactions and hydrogen bonds between neighboring chains [31,32]. Moreover, the incorporation of inclusion complexes increased the free energy value by 8% for P5 and by 11% for P10 as consequence of the polar component contribution in the material surfaces. This result is consistent with the fact that active films were more wettable. This could be attributed to the exposure of hydroxyl groups of β-CD from the inclusion complexes at the surface of active films. Moreover, it is interesting to observe that the dispersive energy for P5 was similar to that for P0, but it drastically reduced for P10. This could be related to the irregularities in the material surface produced by the agglomeration of inclusion complexes, which was probably conducive to water penetration through a capillary effect [27,33].

**Figure A1:**
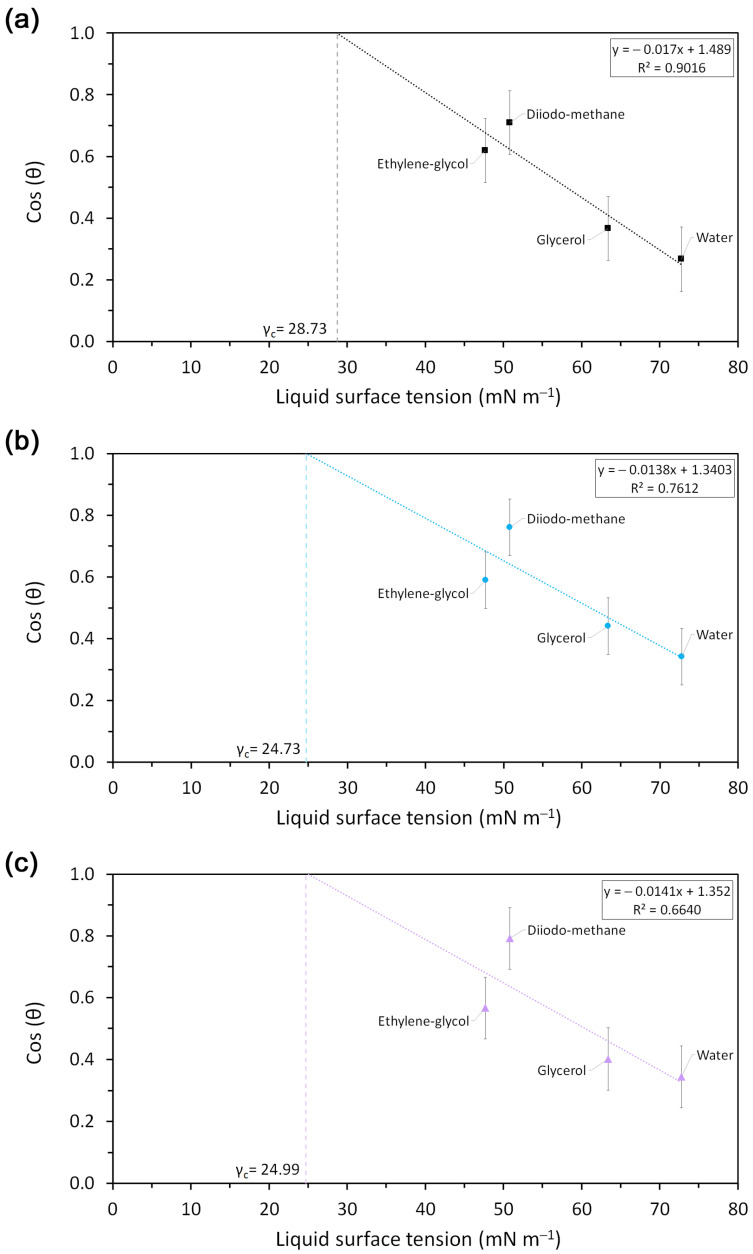
Zisman plot from goniometric measurements for (**a**) P0, (**b**) P5 and (**c**) P10 (experimental values and linear regression).

The critical surface energy (γ_c_) of materials was obtained by the extrapolation to cos θ = 1 in Zisman plots, as shown in Figure A1 of Appendix A. This means that γ_c_ corresponds to a zero wettable energy and, thus, a liquid will be completely spread when its surface tension is equal to or lower than γ_c_ of the material. The γ_c_ for P0, P5 and P10 were 28.7 mN m^−1^, 24.7 mN m^−1^ and 24.9 mN m^−1^, respectively. These values are close to the surface tension value of ethanol (21.4 mN m^−1^), which explains the immediate spread of this solvent over samples during the assay.

### 2.2. Liquid Absorption Capacity

**Figure 2:**
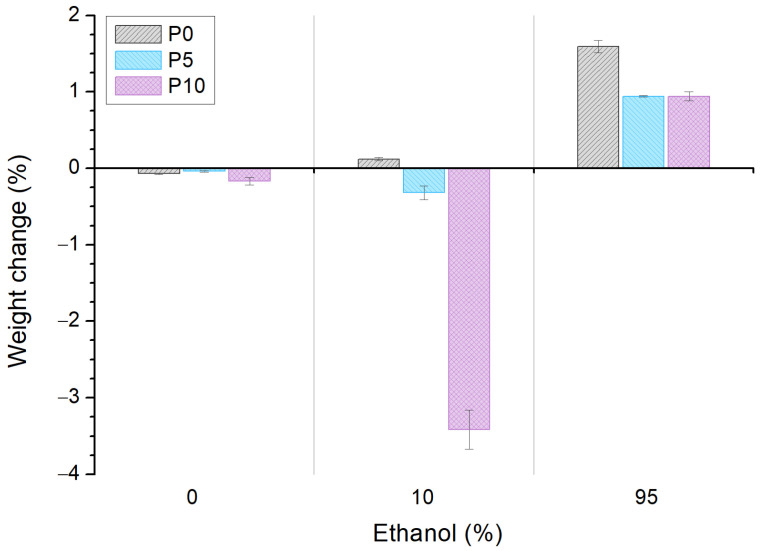
Weight change of poly(lactic acid) (P0) and active films (P5 and P10) after immersion in solutions with different ethanol concentration for 48 h at 25 °C.

Figure 2 shows the weight change of films after their immersion in water, aqueous and fatty simulants (0%, 10% and 95% *v*/*v* ethanol, respectively). All samples slightly decreased their weight after water immersion. However, this change was not significant (<0.2%) and it is explained by the lower water absorption capacity of PLA [34].

In the aqueous simulant, the weight of P0 was almost the same (0.1% change), while a slight (0.3%) and significant (3.4%) decrease in weight was observed for P5 and P10, respectively. The weight loss of active materials was attributed to the highwater-sorption capacity of inclusion complexes, which produced a more wettable surface that triggered AITC release and started the cleavage of the ester bonds [35,36]. Moreover, the significant difference between the P5 and P10 weight loss (10-fold) was related to the high quantity of inclusion complexes in P10 that could increase the free volume in the matrix, favoring the diffusion of the simulant and, therefore, a higher level of polymer hydrolysis. Furthermore, the high weight loss of P10 could be attributed to the release of inclusion complexes to the medium.

A weight increase of >1% was observed for all samples after contact with the fatty simulant. This was attributed to the low difference in the surface tension of films with ethanol, which is absorbed by the matrix [34,37,38]. However, the presence of inclusion complexes in the matrix significantly decreased this behavior, and this was quantitatively similar for P5 and P10. As above, this effect could be related to the release of AITC and the inclusion complexes to the simulant.

### 2.3. Thermal Properties

**Figure 3:**
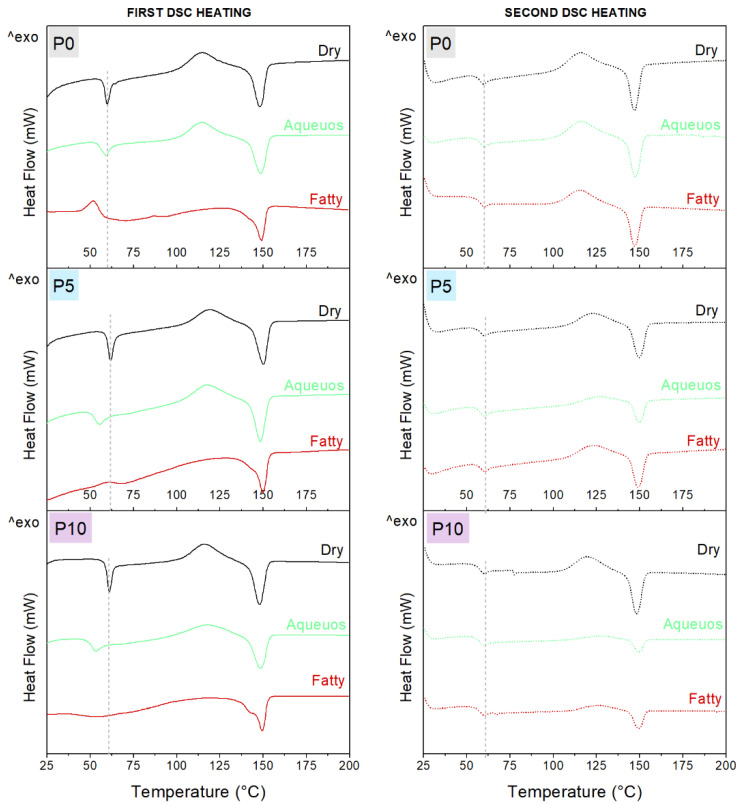
Thermal properties of dry films and after immersion in aqueous and fatty simulants for 48 h at 25 °C.

Changes in the thermal properties of the films following the incorporation of inclusion complexes and contact with food simulants were evaluated by DSC. Figure 3 shows the thermograms, and values are summarized in Table A2 of Appendix B. The cooling scan is not shown, since no thermal transitions were identified in any sample.

During the first and second heating, dry samples (P0, P5 and P10) showed a change in heat flow near to 60 °C, corresponding to the glass transition of PLA (T_g_), followed by an exothermic event between 110 and 120 °C related to cold crystallization (T_cc_) and finally an endothermic peak at 149 °C, specific to PLA melting (T_m_). However, an endothermic peak associated with T_g_ was observed during the first heating. This was related to the mechanical relaxation of the polymer chains in the amorphous zone that could be restricted as result of extrusion and storage of materials. This peak was not observed during the second heating, since polymer chains were reorganized in a controlled way during the cooling [39].

To evaluate the effect of inclusion complexes on the polymer structure, the second DSC heating was considered, while the influence of the simulants on films was evaluated by the first DSC scan. Thus, the transition temperature of PLA was not affected by the incorporation of inclusion complexes. However, the enthalpy associated with cold crystallization (ΔH_cc_) systematically increased with the concentration of the inclusion complexes, probably due to a nucleating effect on the amorphous zone during the DSC heating. In consequence, a significant reduction of the crystallinity of PLA was observed.

Furthermore, a decrease in T_g_ was observed for P0 after immersion in food simulants, especially in the fatty simulant. This effect was attributed to the sorption of water and ethanol in the matrix documented in the Section 2.2, which plasticized the polymer. A similar effect was reported by Xu et al. [34] for casted PLA films. A decrease of T_g_ was also observed for P5 and P10, only after immersion in the aqueous simulant. Moreover, ΔH_cc_ decreased and crystallinity (X_c_) increased in P10 after immersion in this simulant. These changes confirmed the hydrolytic degradation of PLA, which occurred in the amorphous zone and consequently increased the crystallinity [35].

Although the T_g_ of active films was not modified by immersion in fatty simulant, the ΔH_cc_ decreased significantly. This change in ΔH_cc_ was also observed for P0, which confirmed that the phenomenon is only related to the amorphous zone of PLA. Organic solvents such as fatty simulant can trigger solvent-induced crystallization (SIC) of PLA. SIC consists of the absorption of ethanol, which plasticizes and swells the matrix, inducing a rearrangement of amorphous zones or the solubilization of shorter PLA chains, leading to the increase of crystalline zones [36]. Therefore, the significant increase of X_c_ for all materials confirmed that SIC was the driving process in the fatty simulant. However, the differences in T_g_ and ΔH_cc_ between P0 and the active films after immersion in this simulant appear to show that rearrangement of amorphous zones occurred for pure PLA, while the incorporation of inclusion complexes resulted in the direct solubilization of polymer, avoiding the sorption and plasticization steps. This effect could be related to the presence of shorter PLA chains in active films, due to the disruption caused by the inclusion complexes. Indeed, solubilization was more notable in P10, since a double peak was observed during melting after contact with the fatty simulant.

### 2.4. AITC Release

**Figure 4:**
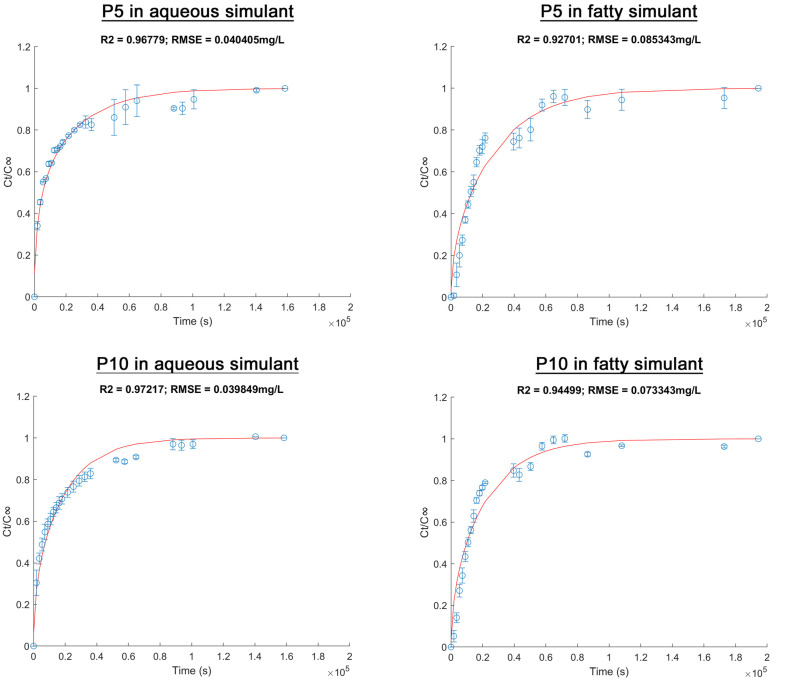
Release of allyl isothiocyanate from poly(lactic acid)/inclusion complex films to food simulants at 25 °C. The red line shows the modeled data based on experimental results (blue points).

The kinetic release of active compounds from materials are shown in Figure 4. In order to normalize the data, experimental points were expressed as the relation between AITC released in a specific period of time and AITC released at equilibrium (*C_t_*/*C_∞_*) vs. time. Diffusion (*D*) and partition (*K_p_*) coefficients were determined from these results fitted by Fickian diffusion model (Equation (5)), where *D* showed the transfer rate of AITC from the materials to the food simulants while *K_p_* (partition coefficient) represented the affinity of AITC for the film or for the simulant [40]. The percentage of released AITC was calculated based on the theoretical initial AITC quantity.

According to Figure 4, the experimental data showed an acceptable adjustment to the Fickian diffusion model (R^2^ > 0.95 y RSME < 0.08). In all cases, a burst release of AITC was observed at the initial stage, followed by equilibrium in the process. However, the release behavior depended on the inclusion complexes’ concentration and the food simulant. Equilibrium for P5 and P10 in the aqueous simulant was reached after 18 h (≈0.6 × 10^5^ s) and 24 h (≈0.9 × 10^5^ s), respectively, whereas it was reached after 30 h (≈1.1 × 10^5^ s) in the fatty simulant for both films. These differences showed that a complex relationship exists. The polymer matrix properties, the inclusion complexes, the active compound and the food simulant type are involved in these relationships. These were explained according to the diffusional parameters and the percentage of active compound released.

Table 2 shows no large differences in the thicknesses of P5 and P10. Therefore, no impact in the diffusivity of AITC was considered due to this factor. Table 2 also shows the diffusional parameters for PLA active films. In both simulants, the *D* coefficient was higher for P10 than for P5. Surface properties, weight change and DSC analysis for P10 showed a higher polar surface, an effective plasticization in the aqueous simulant and a higher solubilization in the fatty simulant. These effects could explain the increase in the AITC diffusion rate through the matrix of P10.

For *K_p_*, a value approximately four times higher was observed for P5 than for P10 in the aqueous simulant. This could demonstrate that released AITC from P5 belongs to the inclusion complexes located at the surface of the films. On the other hand, AITC released from P10 could be from the embedded inclusion complexes, since higher absorption and plasticization was observed for this matrix. Release AITC percentage was twice as high for P10 as for P5 in the aqueous simulant. Furthermore, the *K_p_* value was significantly lower in the fatty than in the aqueous simulant. This means that a higher percentage of AITC was released from the films to the fatty simulant. This is the most common behavior for active materials with compounds derived from essential oils, which is related to the hydrophobic nature of the compound [41,42,43]. In previous sections, it was observed that the surface free energy of materials was dominated by the polar component (*γ_S_^P^* > *γ_S_^D^*). Therefore, high affinity of the PLA matrix with the high concentration ethanol solution was expected. This, in turn, was conducive to the absorption of the fatty simulant, producing a solvent-induced crystallization process. Thus, the solubilization of polymer could favor AITC diffusion and its desorption to the fatty simulant [37,38].

Finally, because the highest value of released AITC percentage (74%) was observed for P5, we can conclude that a maximum of 25% of the initial AITC was lost during the extrusion. This confirms the thermal protection efficacy of AITC, caused by the formation of inclusion complexes with β-CD.

## 3. Materials and Methods

### 3.1. Materials and Reagents

β-cyclodextrin (purity > 98.5%) was obtained from Cyclolab, Ltd. (Budapest, Hungary). The active compound allyl isothiocyanate (AITC > 95%) was purchased from Sigma Aldrich (Santiago, Chile). Poly(lactic acid) resin (PLA) 2003D Ingeo™ was obtained from NatureWorks (Minneapolis, USA). Ethanol (96% (*v*/*v*) purity) for preparing food simulants and for contact angle measurements as well as ethylene glycol, diiodomethane and glycerol were obtained from Sigma Aldrich (Saint-Quentin Fallavier, France).

### 3.2. Inclusion Complexes Preparation

Inclusion complexes β-CD:AITC (1:1 molar ratio) were prepared by a co-precipitation method using the procedure previously reported in Muñoz-Shugulí et al. [15].

### 3.3. Film Preparation

PLA film as a control and active films of PLA with 5 and 10 wt.% of inclusion complexes were prepared by extrusion, based on the fungicidal concentration of β-CD:AITC reported in a previous study [15]. PLA powder resin was dried at 50 °C for 48 h and manually pre-mixed with inclusion complexes. PLA or its mixture was fed into the hooper of a 20 mm co-rotating laboratory twin-screw extruder, Labtech LTE20 (Bangkok, Thailand) and extruded using a temperature profile from 190 °C to 210 °C (feed zone to die zone). Films were collected through a chill roll system (Samutprakarn, Thailand) at 2 m min^−1^ and denoted as P0, P5 and P10 for control and active films, respectively.

### 3.4. Film Characterization

#### 3.4.1. Surface Properties

Contact angle (θ) measurements with five liquids (ethanol, water, ethylene glycol, diiodomethane and glycerol) were carried out using the sessile drop method on a DSA30E Kruss goniometer (Hamburg, Germany) equipped with image analysis software Advance, Kruss GmbH (Hamburg, Germany), according to the method described by Karbowiak et al. [23]. A droplet of each liquid (∼4 µL) was deposited on the film surface with a precision syringe. Image processing and curve fitting from a theoretical meridian drop profile were used to obtain the θ between the baseline of the drop and the tangent at the drop boundary. Four measurements per film were carried out at 25 (±2) °C and RH of 50 (±2)%.

Surface tension (surface free energy) of films (γ_s_) and their polar (γ_s_^P^) and dispersive (γ_s_^D^) components were determined using the Owens–Wendt method as shown in Equation (1) [44]

(1) $$\gamma_{L}\left(1+cos\;\theta \;\right)=2\left(\sqrt{\;\gamma_{S}^{D}\;\gamma_{L}^{D}\;}+\sqrt{\;\gamma_{S}^{P}\;\gamma_{L}^{P}}\right)$$

where *γ_L_*, *γ_L_^D^* and *γ_L_^P^* are the surface tension, dispersive and polar components of the tested liquid expressed in mN m^−1^ (values are shown in Table A1 of Appendix A), while the contact angle (*θ*) is expressed in degrees (°).

Equation (2) is derived by dividing $2\sqrt{\;\gamma_{L}^{D}}$ in Equation (1).

(2) $$\frac{\gamma_{L}\left(1+cos\;\theta \right)}{2\sqrt{\;\gamma_{L}^{D}}}=\sqrt{\gamma_{S}^{p\;}}\;.\sqrt{\frac{\;\gamma_{L}^{p}}{\;\gamma_{L}^{D}}}+\sqrt{\gamma_{S}^{D\;}}$$

Then, the above equation can be represented in the linear form:

*y* = *ax* + *b*

where, $y=\frac{\gamma_{L}\left(1+cos\;\theta \right)}{2\sqrt{\;\gamma_{L}^{D}}}$; $x=\sqrt{\frac{\;\gamma_{L}^{p}}{\;\gamma_{L}^{D}}}$; $a=\sqrt{\gamma_{S}^{p\;}}$ and $b=\sqrt{\gamma_{S}^{D}}$ From this linear presentation, the slope of the graph and the ordinate axis intercept gives, respectively, the polar and dispersive components of the film’s surface free energy.

The Zisman plot for each film was obtained by graphing the cos *θ* vs. *γ_L_* of 5 liquids: ethanol, water, ethylene glycol, diiodomethane and glycerol. The critical surface energy (*γ_c_*) of films was determined by extrapolation of the linear Zisman plot at cos *θ* = 1 [45].

#### 3.4.2. Liquid Absorption Capacity

Changes in the weight of materials immersed in water, 10% and 95% *v*/*v* ethanolic solutions were measured to evaluate the liquid absorption capacity of the materials. Films were cut into pieces of 4 × 5 cm^2^ and weighed (*W_i_*). They were immersed in a glass containing 10 mL of water or ethanolic solution at 25 °C, then the vial was closed and stirred at 150 rpm for 48 h. The simulant remaining on the surface of the film was dried using a paper sheet and the film was weighed again (*W_f_*). The experiment was carried out in triplicate; the liquid absorption capacity was expressed as weight change percentage, and it was calculated by Equation (3).

(3) $$Weight\;change\;\left(\%\right)=\frac{W_{f}-W_{i}}{W_{i}}*100$$

#### 3.4.3. Differential Scanning Calorimetry (DSC)

Dry films and those immersed in ethanol solutions, as detailed in Section 3.4.2, were analyzed by DSC. The assay was performed with 7–8 mg of sample in a sealed aluminum capsule (40 µL capacity). Samples were submitted to a DSC program of three scans, as follows: (i) heating from 0 °C to 200 °C, (ii) cooling from 200 °C to 0 °C and (iii) second heating from 0 °C to 200 °C. All scans were at a rate of 10 °C min^−1^ under a nitrogen atmosphere. Different thermal transitions were obtained as result, and the percentage of crystallinity was calculated using Equation (4) [46].

(4) $$X_{c}\;\left(\%\right)=\frac{\Delta H_{m}-\Delta H_{cc}}{\Delta H_{m}^{0}\;\left(1-X_{IC}\right)}*100$$

where, Δ*H_m_* and Δ*H_cc_* corresponded to the melting and cold crystallization enthalpy of the sample, respectively. $\Delta H_{m}^{0}$ was the theoretical enthalpy for 100% crystalline PLA (93 J g^−1^) and *X_IC_* was the fraction of inclusion complexes incorporated in the film (0.05 for P5 and 0.10 for P10).

### 3.5. Release of AITC from Active Films

#### 3.5.1. Experimental

Film thickness is an important parameter in the diffusivity process of active compounds and, thus, it was initially measured using a Palmer digital micrometer (Barcelona, Spain) at four random positions for each sample. The mean of thickness was used for calculating diffusion and partition coefficients.

The kinetic release of AITC from active films was assessed by total double-side immersion of films in simulants, according to European Regulation (EU) No. 10/2011 [47]. Ethanolic solutions at 10% and 95% *v*/*v* were used as aqueous and fatty food simulants, respectively. Even though isooctane is the recommended substitute for fatty simulant (D2) according to EU regulations, ethanol 95% *v*/*v* has been widely employed to assess the release of compounds from active materials [41,42,43], probably due to the convenience for analytical methods and safety. Pieces of 40 × 5 cm^2^ were immersed into 100 mL of simulant at 25 °C and stirred at 150 rpm to prevent the effect of boundary/stagnant layers at the film interface for non-viscous liquids [48]. The simulant (4 mL) was periodically sampled and the optical density was measured in a Jenway 6305 UV-Visible spectrophotometer (Thermo Fisher Scientific, Waltham, MA, USA) at 245 nm. This maximum wavelength was previously determined for the pure AITC in food simulants. Released active compound was quantified using calibration curves from 0 to 100 mg L^−1^ of AITC in food simulant. The experiments were carried out in triplicate.

#### 3.5.2. Data Modelling and Determination of the Diffusion (*D*) and Partition Coefficient (*K_p_*)

The diffusion coefficient (*D*, m^2^ s^−1^) was determined using a relationship derived from Fick’s second law (transient state) for a unidirectional diffusion in a limited stirred volume [49]. The model considers a limited volume of the film in a limited volume of the simulant for a plane sheet (film). This model, represented by Equation (5), considers that: (a) the initial maximum concentration of the active compound in the film is uniformly distributed at *t* = 0; (b) the initial concentration in the simulant equals zero at *t* = 0; (c) no boundary effect exists (because the medium is perfectly stirred); and (d) the diffusivity is supposed to be neither concentration-dependent nor time-dependent.

(5) $$\frac{C_{t}}{C_{\infty }}=1-\sum_{n=1}^{\infty }\frac{2\alpha \left(1+\alpha \right)}{1+\alpha +\alpha^{2}q_{n}^{2}}\exp\left(-\frac{Dq_{n}^{2}t}{L^{2}}\right)$$

where *C_t_* (mg L^−1^) is the AITC concentration over time in the food simulant; *C_∞_* (mg L^−1^) is the AITC concentration at the equilibrium in the food simulant; *L* corresponds to the half thickness of film; *q_n_* stands for the non-zero positive roots of $tan\;\left(q_{n}\right)=-\alpha q_{n}$ (*n* values ranging from 1 and 6) and $\alpha =\frac{V_{S}}{\left(K_{p\;}\times V_{F}\right)}$; where *V_S_* and *V_F_* correspond to the simulant and film volume (m^3^), respectively. *K_p_* stands for the partition coefficient of the AITC between film and the simulant at equilibrium and was calculated using Equation (6).

(6) $$K_{p}=\frac{C_{F,\infty }}{C_{S,\infty }}$$

where *C_F_*_,∞_ and *C_S_*_,∞_ are the equilibrium concentrations of the AITC (mg L^−1^) in the film and in the food simulant, respectively.

This model was previously adapted and applied by Benbettaieb et al. [50] to experimental release kinetics (up to equilibrium). Modeling was performed using Matlab/Simulink software (MATLAB version R2021a, The MathWorks Inc., Natick, MA, USA). Model calculation was analyzed in triplicate. From each calculation, both the coefficient of determination (R^2^) and the root mean square error (RMSE, mg/L) were determined.

### 3.6. Statistical Analysis

All data were expressed as mean ± standard deviation (SD). Student’s *t*-test or analysis of variance (ANOVA) were carried out for comparisons, using InfoStat software (Córdova, Argentine) with a significance value of α = 0.05, followed by a Tukey test to determine differences, if necessary.

## 4. Conclusions

In this study, active films based on PLA loaded with β-CD:AITC inclusion complexes were developed. In addition, changes in the material properties during the release of AITC to food simulants were assessed.

The surface properties of PLA film were related to the absorption behavior of different simulants. Since the PLA surface was wettable, it was susceptible to hydrolysis by immersion in an aqueous simulant. On the contrary, the proximity of the surface free energy (γ_s_) of PLA to the ethanol surface tension caused the film to easily absorb the fatty simulant. Thus, significant plasticization and solubilization of the polymer matrix was produced.

The incorporation of inclusion complexes β-CD:AITC in PLA resulted in films with a more wettable and polar surface. This effect encouraged the diffusion of water and aqueous simulant through the matrix, favoring polymer hydrolysis, especially when inclusion complexes at 10 wt.% were used. Interestingly, the incorporation of β-CD:AITC in PLA films avoided absorption and plasticization of the matrix when immersed in the fatty simulant, but not solubilization. Therefore, it was concluded that change in the properties of PLA/β-CD:AITC films on contact with food simulants is strongly related to the concentration, water-sorption capacity and solubility of the inclusion complexes.

These complicated relationships also took place in the release of AITC to food simulants. Thus, higher concentrations of inclusion complexes in PLA increased the AITC release rate (*D* coefficient). Moreover, even though inclusion complexes increased the interaction of PLA with the aqueous simulant, the effect of polymer solubilization by the fatty simulant and its affinity with AITC favored the release of the active compound (lower *K_p_*).

Understanding this phenomenon could facilitate the development of more appropriate PLA/β-CD:AITC films for the packaging of high-fat fruits and vegetables such as avocadoes, olives, coconuts, seeds and nuts. However, it was demonstrated that ethanol causes great changes in the properties of the PLA matrix and favors AITC release. Therefore, it is recommendable that attention is paid to evaluation of active compound release from PLA to 95% ethanol as a fatty simulant. Although this could be addressed by using alternative fatty simulants such as isooctane and 50% ethanol, more research about the relationship between these simulants, the PLA matrix, inclusion complexes and active compound release is needed.

## Figures and Tables

**Table 1:** Contact angle (θ) of poly(lactic acid) (P0) and active films (P5 and P10).

Material	θ_w_ (°)	θ_EG_ (°)	θ_DM_ (°)	θ_G_ (°)
P0	74.5 ± 1.4 ^a^	51.7 ± 2.0 ^b^	44.8 ± 0.4 ^a^	68.5 ± 3.9 ^a^
P5	70.0 ± 1.3 ^b^	53.9 ± 0.6 ^a,b^	40.4 ± 1.7 ^b^	63.9 ± 2.0 ^a^
P10	69.9 ± 0.8 ^b^	55.5 ± 0.9 ^a^	37.7 ± 1.7 ^c^	66.3 ± 1.6 ^a^

Means with different lowercase letters (a, b, c) show statistically significant differences between the films (*p* < 0.05) according to ANOVA and Tukey’s tests.

**Table 2:** Kinetic parameters for the release of allyl isothiocyanate from active poly(lactic acid) films to different food simulants, obtained from Fickian model fitting.

	Ethanol in Simulant	P5	P10
	(% *v*/*v*)		
Thickness (µm)		83.6 ± 5.7	96.6 ± 5.6
*D* (m² s^−1^) × 10^12^			
In aqueous simulant	10	4.6 ± 1.0 ^b,B^	14.4 ± 0.2 ^a,A^
In fatty simulant	95	8.1 ± 1.4 ^a,B^	11.6 ± 0.1 ^a,A^
*K_p_*			
In aqueous simulant	10	140.2 ± 4.9 ^a,A^	33.5 ± 1.1 ^a,B^
In fatty simulant	95	16.48 ± 1.7 ^b,B^	27.7 ± 2.4 ^b,A^
Released AITC (%)			
In aqueous simulant	10	27.6 ± 1.5 ^b,B^	57.4 ± 1.7 ^b,A^
In fatty simulant	95	74.1 ± 5.0 ^a,A^	65.7 ± 1.0 ^a,B^

Means with different lowercase letters (a, b) within a column show statistically signficant differences between the simulants (*p* < 0.05); means with different capital letters (A, B) within a row show statistically signficant differences between films (*p* < 0.05), according to Student’s-*t* tests.

## Data Availability

The data presented in this study are available on request from the corresponding authors.

## Sample Availability

Samples of the compounds are not available from the authors.

## Appendix A

**Table A1:** Surface tension components of liquids.

Liquid	γ_L_ (mN m^−1^)	γ_L_^P^ (mN m^−1^)	γ_L_^D^ (mN m^−1^)
Water	72.8	51.0	21.8
Ethanol	21.4	18.8	2.6
Ethylene glycol	48.8	32.8	16.0
Glycerol	63.4	26.4	37.0
Diiodomethane	50.8	2.3	48.5

γ_L_ → surface tension, γ_L_^P^ → polar component, γ_L_^D^ → dispersive component. Values obtained from Diversified Enterprises (2014) [51].

## Appendix B

**Table A2:** Results of DSC analysis of poly(lactic acid) (P0) and active films (P5 and P10) before and after immersion in food simulants at 25 °C for 48 h.

**First Heating**				
	**Ethanol in Simulant**	**P0**	**P5**	**P10**
	**(% *v*/*v*)**			
T_g_ (°C)				
Dry film	-	59.18 ± 1.00	60.58 ± 0.01	60.19 ± 0.11
In aqueous simulant	10	56.29 ± 0.87	53.15 ± 0.26	51.54 ± 0.16
In fatty simulant	95	53.14 ± 1.69	63.51 ± 0.14	58.07 ± 1.45
T_cc_ (°C)				
Dry film	-	114.73 ± 0.51	118.89 ± 0.16	116.40 ± 0.61
In aqueous simulant	10	113.65 ± 0.71	118.25 ± 0.33	117.51 ± 0.31
In fatty simulant	95	123.55 ± 2.83	118.42 ± 1.50	-
ΔH_cc_ (J g^−1^)				
Dry film	-	23.40 ± 0.23	23.26 ± 0.15	27.42 ± 0.14
In aqueous simulant	10	23.45 ± 0.34	22.95 ± 0.48	18.57 ± 1.38
In fatty simulant	95	5.82 ± 0.01	2.38 ± 0.26	-
T_m_ (°C)				
Dry film	-	148.35 ± 0.18	149.75 ± 0.36	148.07 ± 0.16
In aqueous simulant	10	147.50 ± 1.66	148.84 ± 0.47	149.14 ± 0.91
In fatty simulant	95	149.39 ± 0.63	149.75 ± 0.18	149.86 ± 0.54
ΔH_m_ (J g^−1^)				
Dry film	-	31.33 ± 0.53	29.35 ± 0.62	30.44 ± 0.23
In aqueous simulant	10	32.45 ± 0.25	30.66 ± 0.63	29.71 ± 0.82
In fatty simulant	95	23.41 ± 0.04	27.03 ± 1.19	27.75 ± 2.51
X_c_ (%)				
Dry film	-	8.52 ± 0.33	6.89 ± 0.86	3.61 ± 0.44
In aqueous simulant	10	9.68 ± 0.09	8.72 ± 0.17	13.32 ± 0.67
In fatty simulant	95	18.92 ± 0.04	27.91 ± 1.05	33.15 ± 3.00
**Second Heating**				
	**Ethanol in Simulant**	**P0**	**P5**	**P10**
	**(% *v*/*v*)**			
T_g_ (°C)				
Dry film	-	57.53 ± 0.08	58.74 ± 0.15	57.96 ± 0.15
In aqueous simulant	10	58.33 ± 0.28	57.80 ± 0.01	58.39 ± 0.12
In fatty simulant	95	58.53 ± 0.018	58.57 ± 0.05	57.09 ± 0.22
T_cc_ (°C)				
Dry film	-	115.39 ± 0.04	122.62 ± 0.29	119.65 ± 0.02
In aqueous simulant	10	115.73 ± 0.11	125.97 ± 1.19	127.09 ± 0.64
In fatty simulant	95	115.60 ± 0.46	123.29 ± 0.40	126.71 ± 0.37
ΔH_cc_ (J g^−1^)				
Dry film	-	18.36 ± 0.24	20.29 ± 0.04	25.05 ± 0.07
In aqueous simulant	10	23.49 ± 0.41	10.05 ± 1.84	7.95 ± 1.44
In fatty simulant	95	24.18 ± 0.48	17.52 ± 0.16	8.40 ± 0.13
T_m_ (°C)				
Dry film	-	147.24 ± 0.28	149.34 ± 0.28	148.15 ± 0.20
In aqueous simulant	10	147.15 ± 0.47	149.55 ± 0.27	150.02 ± 0.66
In fatty simulant	95	147.28 ± 0.25	149.09 ± 0.06	149.55 ± 0.25
ΔH_m_ (J g^−1^)				
Dry film	-	25.97 ± 0.41	20.85 ± 0.13	26.27 ± 0.11
In aqueous simulant	10	28.17 ± 0.72	14.24 ± 2.45	11.64 ± 1.32
In fatty simulant	95	25.96 ± 0.14	18.93 ± 0.25	10.73 ± 0.23
X_c_ (%)				
Dry film	-	8.18 ± 0.18	0.64 ± 0.18	1.46 ± 0.05
In aqueous simulant	10	5.03 ± 1.22	4.74 ± 0.70	4.40 ± 0.14
In fatty simulant	95	1.92 ± 0.67	1.60 ± 0.46	2.79 ± 0.42

T_g_ → glass transition temperature, T_cc_ → cold crystallization temperature, ΔH_cc_ → cold crystallization heat, T_m_ → melting temperature and ΔH_m_ → melting heat. Values are the mean of two replicates.
